# Is it feasible to treat unstable traumatic spondylolisthesis of the axis via posterior fixation without fusion?

**DOI:** 10.1186/s12891-023-06233-z

**Published:** 2023-02-13

**Authors:** Jian Zhang, Guangzhou Li, Qing Wang

**Affiliations:** Department of Orthopaedics, the Affiliated Hospital of Southwest Medical University, No. 25 Taiping Street, Luzhou, Sichuan Province 646000 China

**Keywords:** Traumatic spondylolisthesis of the axis, Posterior fixation, Radiographic outcomes, Clinical outcomes, Spontaneous fusion

## Abstract

**Background:**

Few studies reported treatment of unstable traumatic spondylolisthesis of the axis using posterior fixation without fusion. The aim of this study was to evaluate the results and feasibility of posterior fixation without fusion in treating unstable traumatic spondylolisthesis of the axis.

**Methods:**

Eleven patients with traumatic spondylolisthesis of the axis were included in this study, and posterior fixation without fusion using screw-rod system was performed for them. The clinical outcomes were assessed using the Visual Analog Scale (VAS), the Neck Disability Index (NDI), and the Odom’s grading system. Plain radiography was used to measure the displacement and angulation of C2-C3, and cervical lordosis. Plain radiography and computed tomography were also used to observe the bony fusions of fracture lines and postoperative spontaneous fusion of C2-C3.

**Results:**

The mean follow up time was 24.6 months (range, 12–72 months). The VAS and NDI scores were significantly improved at the final follow-up compared with those before operation (*P* < 0.05), and according to Odom’s criteria, 90.9% (10/11) of patients rated their level of satisfaction as excellent or good. The angulation and displacement of C2-C3, and cervical lordosis were significantly improved after operation compared with those before operation (*P* < 0.05), and at the final follow-up, and these radiological parameters were maintained. All patients achieved solid bony fusions of fracture lines. No operative segment instability was found in all patients during the follow-up period. Spontaneous fusion at bilateral C2-C3 facet joints was found in 11 cases, and anterior and/or posterior bony bridge of intervertebral bodies at C2-C3 was found in 9 cases.

**Conclusions:**

Posterior fixation without fusion may be a feasible and effective option for unstable traumatic spondylolisthesis of the axis.

## Background

The traumatic spondylolisthesis of the axis, also known as axis ring fracture or Hangman fracture, is the second common type of C2 fracture [1–4]. It is widely accepted that type II, type IIa, and type III lesions in Levine-Edwards classification usually are unstable fractures and should be treated with operative treatment [2–4]. However, the choice of surgical methods for traumatic spondylolisthesis of the axis is still controversial [4–7]. The available options of surgical methods include anterior C2-C3 discectomy with fusion, posterior C2-C3 fixation and fusion, posterior C2 pedicle screw alone, and combined anterior & posterior fixations and fusion, and each of them has its own advantages and disadvantages [4, 6]. Among the different approaches, anterior or posterior C2-C3 fixation and fusion is the most widely used one [4, 6, 7].

Both anterior and posterior approaches can effectively treat unstable traumatic spondylolisthesis of the axis, resulting in a high rate of fusion of fracture lines [4, 6]. No matter anterior or posterior operation, segmental fixation with fusion using autogenous bone graft seems to be the “gold standard” [8, 9]. However, autogenous bone graft by iliac crest or others may have many complications, such as pain in the donor area, infection, nerve injury, abdominal organ hernia through the donor area, and so on [10]. Meanwhile, the usage of allograft bone or artificial bone graft has its associated risks, and increases the cost of healthcare [8, 10]. In fact, the phenomenon of the postoperative spontaneous bone fusion of C2-C3 does exist, and spontaneous bone fusion of C2-C3 has been reported even in patients receiving conservative treatment [2]. To our knowledge, there is no research report describing the postoperative spontaneous bone fusion in detail. Meanwhile, few studies reporting treatment of unstable traumatic spondylolisthesis of the axis using posterior fixation without fusion have been published until now. Therefore, this study aimed to evaluate the results and feasibility of posterior fixation without fusion in treating unstable traumatic spondylolisthesis of the axis.

## Methods

### Patient population

This was a retrospective clinical study. Inclusion criteria were as follows: (1) patients with Levine-Edwards type II, IIa, III traumatic spondylolisthesis of the axis [2], and type-I fracture with neurological impairment; (2) posterior fixation without bone graft; (3) a minimum of 1-year follow-up; (4) the clinical and imaging data were complete. Patients who underwent direct repair of C2 ring with only posterior C2 pedicle screws were excluded. The study protocol was approved by the Ethics Committee of authors’ affiliated institution. All patients included in this study provided a written informed consent.

According to the inclusion and exclusion criteria, 11 patients treated with posterior surgery in our hospital from 2011 to 2017 were included in this study (Table 1). There were 9 males and 2 females, with the mean age of 34.4 years (range, 15–56). The causes of injuries were traffic accident in 7 cases, and high fall in 4 cases. According to Levine-Edwards classification, there were 1 case of type I fracture with neurological impairment, and 6 case of type II, 3 cases IIa, and 1 case of III lesions [2]. The severity of neurological deficit was assessed by the American Spinal Injury Association (ASIA) scale, and 3 cases were found with neurological impairment, including 2 cases classified as ASIA D and 1 case as ASIA C [11].

**Table 1:** Demographics and summary of 11 patients

**Patient**	**Age (Years)**	**Sex**	**Cause of Injury**	**Levine-Edwards** **Classification**	**ASIA**	**Final follow-up (months)**
1	51	M	Fall	II	D	13
2	23	M	MVA	I	D	12
3	15	M	MVA	IIa	E	18
4	26	F	MVA	IIa	E	18
5	51	M	MVA	II	E	24
6	40	F	Fall	II	E	24
7	56	M	MVA	II	E	13
8	41	M	Fall	II	C	72
9	15	M	MVA	III	E	12
10	33	M	MVA	II	E	30
11	27	M	Fall	IIa	E	35

*ASIA* American Spinal Injury Association scale, *M* male, *F* female, *MVA* motor vehicle accident

### Procedures

#### Preoperative Care

After admission, the patient was immobilized by a Philadelphia collar, and received radiological examinations, including X-ray, CT and 3D reconstruction images, and magnetic resonance imaging of the cervical spine. The development and course of vertebral artery were observed, and C1, C2, and C3 pedicle screw channels were designed by CT and 3D reconstruction images. Usually, patients were treated with surgery at 2–3 days after admission.

#### Surgical Technique

All the operations were performed under general endotracheal anesthesia, and the patients were placed in a prone position on the operating table, with continuous 4-5 kg skull traction to maintain the mild flexion of the cervical spine. Among the 11 patients, segments of posterior screw fixation were described as follows: 8 cases were treated with posterior C2-C3 pedicle screw fixation; 1 case was treated with C2 pedicle screw and C3 lateral mass screw fixation, because of C3 pedicle screws could not be safely placed; 2 cases were treated with C1-C3 pedicle screw fixation (posterior C2 and C3 pedicle screw fixation, plus bilateral C1 pedicle fixation), because of obvious displacement of fracture of superior articular process in C2.

- I. A standard midline exposure was carried out until C2-C3 posterior elements were clearly exposed.
- II. In C2, the entry point was decorticated by a burr or awl to create a pilot hole, and the trajectory of pedicle screw was based on the pre-operative CT finding. And then an awl with different length was advanced slowly, and the tract should be parallel to the medial edge of the pedicle. A ball-tipped feeler was used to confirm that there was no cortical breach, and a tap was used to prepare the tract for the screw. Then, C2 pedicle screws were instrumented.
- III. In C3, pedicle or lateral mass screws were placement. And C1 pedicle screws were also placed when it was necessary (two patients in this group). Then, two connecting rods were installed in bilateral sides.
- IV. The reduction of translation and angulation of C2-C3 was achieved by manipulating the rod with compression or distraction. If the reduction of translation and angulation of C2-C3 was satisfactory by the C-arm fluoroscopic, and then fixation device was locked. No autologous or allogous bone grafts were used.

The incision was closed, and a drainage tube was routinely placed. After operation, antibiotics were used prophylactically twice. The drainage tube was removed within 24–48 h after operation, and then patients were encouraged to ambulate with cervical collars.

### Clinical and radiographical evaluations

The clinical data and radiographical outcomes were routinely collected preoperatively and at routine postoperative intervals of 1 week, 3 months, 6 months, 12 months, and at the final follow-up. The visual analogue scale (VAS) scores were used to evaluate the severity neck pain, and the neck disability index (NDI) scores were used to evaluate the function of the neck [12, 13]. And patient satisfaction with the surgery was assessed using Odom’s grading system [13].

**Fig. 1:**
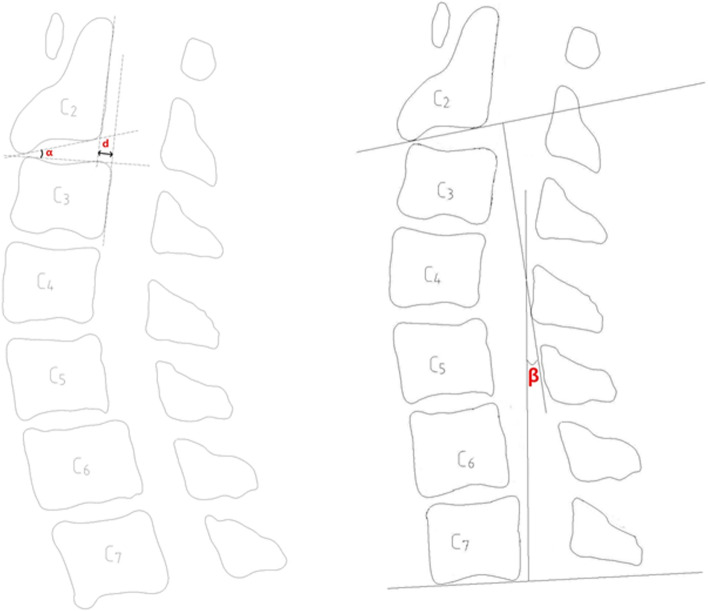
Left–A schematic diagram showing that displacement of C2-3 is measured as the distance between lines drawn parallel to the posterior margins of the C2 and C3 bodies at the level of the disc space (d: displacement of C2/3), and angulation of C2-3 is measured as the angle formed by lines drawn along the inferior endplate of the C2-C3 vertebrae (α: angulation of C2/3). Right–-A schematic diagram showing that cervical lordosis is measured (β: Cobb angle of C2-C7)

Lateral-view radiographs were used to measure the displacement and angulation of C2-C3, and cervical lordosis were measured using the Cobb method (Fig. 1) [13]. Plain radiography and computed tomography were also used to observe the bony fusions of fracture lines and postoperative spontaneous fusion of C2-C3 [2, 14, 15].

### Statistical analysis

Statistical analysis was performed in SPSS statistical software (IBM SPSS Statistics for Windows, Version 24.0; IBM Corp., Armonk, NY). The results are presented as the means standard deviations, and a paired t test was used to compare between preoperative and postoperative parameters. *P* < 0.05 was considered to be indicative of statistical significance.

## Results

All the operations of 11 patients were completed successfully, and there were no perioperative complications. The mean follow-up period was 24.6 months (range, 12–72 months). VAS score and NDI score before operation, after operation, and at the final follow-up were shown in Table 2. The VAS and DNI scores were significantly improved after operation and at the last follow-up (*P* < 0.05, respectively), and the VAS and DNI scores at the final follow-up period were further improved as compared with those after operation (*P* < 0.05, respectively). According to Odom’s criteria, 10/11 (90.9%) patients rated their level of satisfaction as excellent or good at the final follow-up. There was no neurologic deterioration in any patient. Neurologic evaluation showed that 1 patient with ASIA C improved to ASIA E, and 2 patients with ASIA D also improved to ASIA E at the last follow-up.

**Table 2:** VAS and NDI scores of patients preoperatively and during follow-up

	**Pre- operative**	**Post- operative**	**Final follow-up**	**Statistical value**					
				**T1**	**P1**	**T2**	**P2**	**T3**	**P3**
VAS	4.9 ± 0.2	1.7 ± 0.6	1.1 ± 0.2	26.1	0.000	21.0	0.000	4.18	0.002
NDI	28.9 ± 1.5	7.1 ± 0.5	3.1 ± 0.5	14.5	0.000	15.7	0.000	7.20	0.000

*Note*: T1 and P1 indicating postoperative index compared with preoperative index; T2 and P2 indicating final follow-up postoperative index compared with preoperative index; T3 and P3 indicating final follow-up postoperative index compared with postoperative index

All the 9 patients with C2-C3 fixation did not complain disability of neck activity during postoperative follow-up, and among them 8 patients underwent operations of removal of implants 1–2 years after surgery. While one patient who reported no neck discomfort refused to undergo operations of removal of implants, and the internal fixation was not removed within 6 years of follow-up. Two patients with C1-C3 fixation complained of obvious limitation of neck movement after operation, and with removal of implants 1 year after operation, they reported that the neck movement was significantly improved after the final follow-up period.

The imaging data before operation, after operation and the final follow-up period were shown in Table 3. After operation and at the final follow-up, the angulation and displacement of C2-C3 were significantly improved as compared with those before operation (*P* < 0.05). At the final follow-up, the angulation and displacement of C2-C3 were slightly lost compared with those after operation, but the difference was not statistically significant (*P* > 0.05). The changes of cervical lordosis (C2-C7 Cobb) angle after operation and the final follow-up showed a similar trend with the angulation and displacement of C2-C3.

**Table 3:** The radiological data of patients preoperatively and during follow-up

	**Pre- operative**	**Post- operative**	**Final follow-up**	**Statistical value**					
				**T1**	**P1**	**T2**	**P2**	**T3**	**P3**
Angulation of C2/3(°)	8.4 ± 1.8	1.7 ± 0.5	2.6 ± 0.9	4.35	0.001	3.98	0.003	-1.61	0.138
Displacement of C2/3(mm)	4.1 ± 0.3	0.55 ± 0.3	0.7 ± 0.3	9.09	0.000	7.78	0.000	-1.49	0.167
Cobb’s angle of C2/7(°)	17.9 ± 1.6	23.0 ± 1.6	21.2 ± 1.8	-3.81	0.003	-2.44	0.035	1.79	0.103

*Note*: T1 and P1 indicating postoperative index compared with preoperative index; T2 and P2 indicating final follow-up postoperative index compared with preoperative index; T3 and P3 indicating final follow-up postoperative index compared with postoperative index

**Fig. 2:**
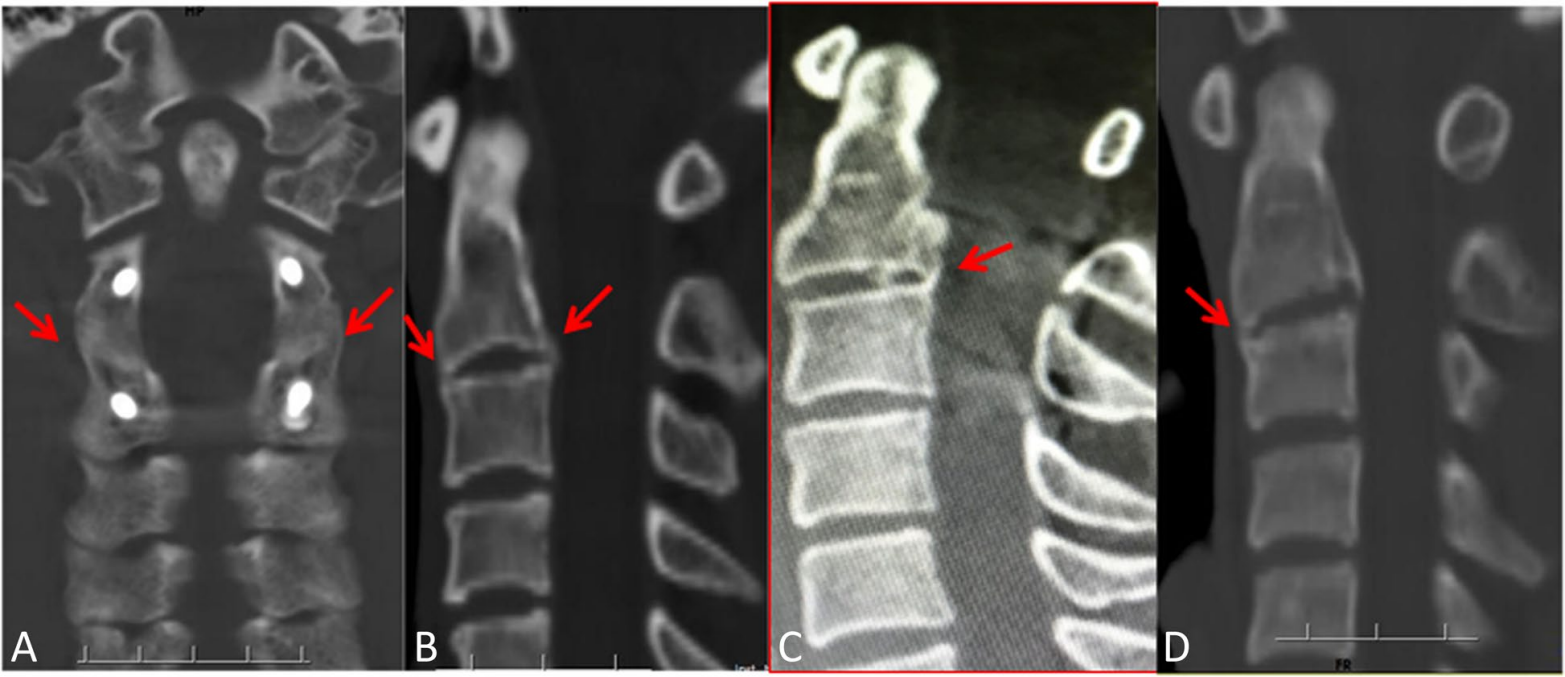
CT scans showing the spontaneous fusion of C2-C3: **A** spontaneous fusion of at bilateral C2-C3 facet joints shown by the red arrow; **B** both anterior and posterior bony bridge at C2-C3 of the intervertebral bodies shown by the red arrow; **C** posterior bony bridge at C2-C3 of the intervertebral bodies shown by the red arrow; **D** anterior bony bridge at C2-C3 of the intervertebral bodies shown by the red arrow

All the fracture lines of axis ring in 11 patients healed well during the last follow-up. Except for a breakage C2 pedicle screw on the right side was found in 1 patient (1 year after operation), and there was no failure of implants in the remaining patients, and no operative segmental instability was found in all patients during the follow-up period. Spontaneous fusion at bilateral C2-C3 facet joints was found in 11 patients, and both anterior and posterior bony bridge of intervertebral bodies at C2-C3 was found in 2 patients, a posterior bony bridge in 6 patients, and an anterior bony bridge in 1 patient (Fig. 2). And no formation of anterior or posterior bony bridge of intervertebral bodies at C2-C3 was found in 2 patients.

### Representative cases

**Fig. 3:**
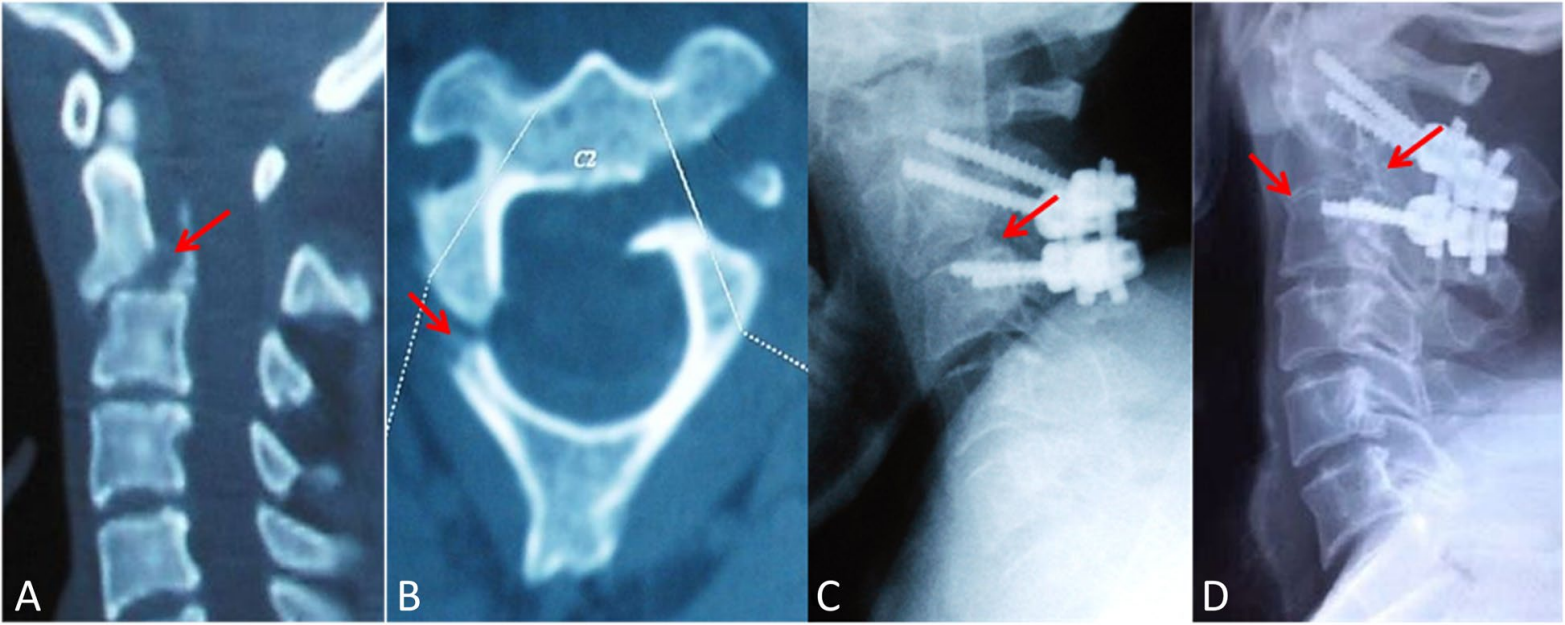
A 41-year-old man (case number 8) suffered from traumatic spondylolisthesis of the axis (Levine-Edwards type II) and underwent surgery of posterior fixation without fusion: **A** CT scan of middle sagittal plane at the time of admission showing obvious angulation and displacement between C2-C3; **B** Axial CT scan showing atypical fracture (one fracture line through posterior cortex of C2 on the left side with contralateral lamina fracture); **C** Lateral x-ray on postoperative 7th day showing that posterior C2-C3 pedicle screw fixation without bone graft was performed; **D** Lateral x-ray at 72 months after operation showing spontaneous fusion at bilateral C2-C3 facet joints and both anterior and posterior bony bridge of intervertebral bodies at C2-C3

**Fig. 4:**
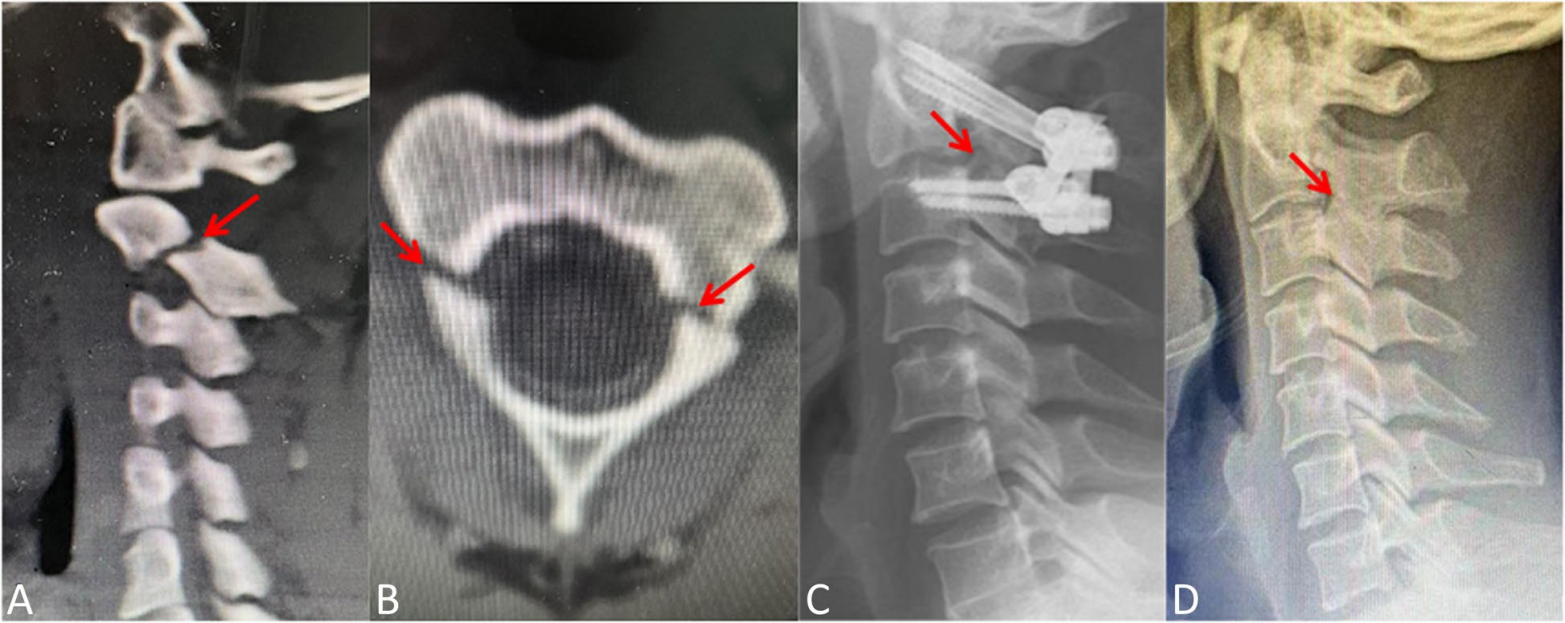
A 33-year-old man (case number 10) suffered from traumatic spondylolisthesis of the axis (Levine-Edwards type II), and he also underwent surgery of posterior fixation without fusion: **A** CT scan of parasagittal plane at the time of admission showing pars fracture of C2 on left side; **B** Axial CT scan showing bilateral pars fractures of C2; **C** Lateral x-ray on postoperative 7th day showing posterior C2-C3 pedicle screw fixation without bone graft was performed; **D** Lateral x-ray at 30 months following the initial surgery showing spontaneous fusion at bilateral C2-C3 facet joints (The patient underwent operation of removal of implants at 24 months after fixation surgery)

Representative cases are illustrated in Fig. 3A–D and Fig. 4A–D.

## Discussion

Murphy et al. [6] analyzed the literature and concluded that both an anterior and a posterior approach could result in a high rate of fusion in the management of traumatic spondylolisthesis of the axis, and neither approach seemed to be superior. However, no matter anterior or posterior operation, most of papers included in Murphy et al.’ review used segmental fixation with fusion using bone graft for traumatic spondylolisthesis of the axis [6]. To our best knowledge, this is the first study focusing on the treatment for unstable traumatic spondylolisthesis of the axis with posterior segmental fixation without fusion and describing the details of postoperative spontaneous fusion at C2-C3 level. Our study showed that management of unstable traumatic spondylolisthesis of the axis using posterior fixation without fusion could result in favorable clinical and radiological outcomes. Most importantly, the results of this study showed the postoperative spontaneous fusion of C2-C3 was very common, demonstrating that it could avoid the need of autogenous bone graft.

The recognized goals of surgical treatment of unstable traumatic spondylolisthesis of the axis are reduction, stabilization, and maintenance of alignment of the cervical spine [13]. Surgery could be performed from anterior, posterior, or combined anteroposterior approaches, and each approach has its unique advantages and disadvantages [12, 13, 16]. Bone graft fusion is usually used in the operation of unstable traumatic spondylolisthesis of the axis to achieve medium-and long-term stability. And posterior fixation and fusion is one of common used techniques for unstable traumatic spondylolisthesis of the axis [2, 4, 6, 16]. In 1985, Levine et al. [2] first noticed the phenomenon of spontaneous fusion of C2-C3 in 4 patients with Levine-Edwards type II injury. Unfortunately, such phenomenon has not attracted the attention of researchers until now. In 2019, Ma X et al. [17] reported their experience with 9 patients using posterior fixation and non-fusion for fresh type II and type IIA traumatic spondylolisthesis of the axis, but the aim of their study was to preserve the motion of C2-C3 after implant removal, neglecting the phenomenon of spontaneous fusion of C2-C3. Recent literature also supports the feasibility of posterior fixation without fusion for unstable traumatic spondylolisthesis of the axis [18].

In our opinion, it is feasible to manage the unstable traumatic spondylolisthesis of the axis using posterior fixation without bone grafting. Firstly, in theory, unstable traumatic spondylolisthesis of the axis can be turned into a stable fracture (similar to “Levine-Edwards type I fracture”) by posterior fixation, and a stable fracture does not need bone grafting [2, 6, 12]. Secondly, if the spontaneous fusion of C2-C3 is common, it certainly maintains the medium-and long-term stability of C2-C3. In this study, all the 11 patients achieving spontaneous fusion at posterior facet joint between the C2/3 level, and 9 of 11 patients with an anterior, posterior, or both anterior and posterior bony bridge at C2-C3 level, which means that posterior fixation without bone graft can guarantee the medium-and long-term stability of C2-C3. Thirdly, this study suggests that posterior fixation without bone graft fusion can achieve favorable clinical outcomes in the treatment of unstable traumatic spondylolisthesis of the axis, and the angulation and displacement of C2-C3 can be satisfactorily corrected, and the normal curvature of the cervical vertebra can be satisfactorily maintained. Lastly, recent studies support the feasibility of posterior fixation without fusion for unstable traumatic spondylolisthesis of the axis [17, 18]. In addition, Kahanovitz N et al. [19] have found that internal fixation of the spine without arthrodesis in animal experiment or patients could cause facet joints changes, including osteophyte formation, subchondral remodeling, and so on, which might provide an important clue in spontaneous fusion of C2-C3 of our patients treated with posterior fixation without bone grafting. Therefore, we cautiously suggest that the posterior fixation without fusion is a safe and feasible method for the treatment of unstable traumatic spondylolisthesis of the axis, considering the limitation of this present study by its retrospective nature and small number of cases.

The advantages of posterior fixation without fusion for the treatment of unstable traumatic spondylolisthesis of the axis are obvious, and it avoids the need of harvesting of iliac bone, allograft bone, or artificial bone graft and their associated risks, and reduces the cost of surgery. Posterior fixation and fusion also has the advantages of reliable fixation and strong stability using pedicle screw, which provides superior biomechanical stability compared with anterior plate [16]. In addition, it is easier for most spinal surgeons to master the techniques of posterior cervical surgery. Although it has been reported that posterior pedicle screw placement has a high risk of injuries to important structures such as vertebral artery and nerve, careful preoperative preparation, including observing vertebral artery course and pedicle morphology, using surgical navigation equipment, and so on, can avoid or reduce these risks [9, 16, 20].

In this study, for patients who need C1-C3 internal fixation, such as obvious displacement of C2 superior articular process fracture, posterior C2-C3 pedicle screw plus temporary fixation of bilateral C1 pedicle fixation should be considered [20]. And satisfactory cervical range of motion can still be obtained, when implant is removed.

Some study limitations should be mentioned. First, this study was a single-center retrospective study with a small number of cases. Second, the cases in this study were not compared with the cases of fusion. We are collecting patients with a larger sample size, and collecting data from patients underwent posterior fixation with simultaneous bone graft fusion, and we will conduct a compared study in medium-and long-term reports.

## Conclusion

For unstable traumatic spondylolisthesis of the axis, posterior fixation without fusion results in favorable clinical and radiological outcomes. Most importantly, since the postoperative spontaneous fusion of C2-C3 is common after posterior fixation, it can avoid complications such as donor site pain. Considering the limitation of this present study by its retrospective nature and small number of cases, we cautiously suggest that the posterior fixation without fusion may be a feasible and effective option for unstable traumatic spondylolisthesis of the axis.

## Data Availability

The datasets used and/or analyzed during the current study are available from the corresponding authors on reasonable request.

## Abbreviations

VAS: Visual Analog Scale
NDI: Neck Disability Index
CT: Computed tomography
ASIA: American Spinal Injury Association scale
